# Supplementary material for: Synthesis and Characterization of Mannosylated Formulations to Deliver a Minicircle DNA Vaccine
Source: Pharmaceutics. 2021 May 7;13(5):673. doi: 10.3390/pharmaceutics13050673 (PMC8150592; doi:10.3390/pharmaceutics13050673)
Supplement: Supplementary file 1 [file pharmaceutics-13-00673-s001.zip › pharmaceutics-1194450-supplementary.pdf]

pharmaceutics

Supplementary Materials: Synthesis and Characterization of Mannosylated Formulations to Deliver a Minicircle DNA Vaccine

Ana Sofia Serra [1], Dalinda Eusébio [1], Ana Raquel Neves [1], Himanshu Bhatt [2], Swati Biswas [2], Diana Costa [1,*] and Ângela Sousa [1,*]

Citation: Serra, A.S.; Eusébio, D.; Neves, A.R.; Albuquerque, T.; Bhatt, H.; Biswas, S.; Costa, D.; Sousa, Â. Synthesis and characterization of mannosylated formulations to deliver a minicircle DNA vaccine. *Pharmaceutics* **2021**, *13*, 673. https://doi.org/10.3390/pharmaceutics13050673

Academic Editor: Satoshi Uchida

Received: 7 April 2021
Accepted: 4 May 2021
Published: 7 May 2021

Publisher's Note: MDPI stays neutral with regard to jurisdictional claims in published maps and institutional affiliations.

Copyright: © 2021 by the author. Licensee MDPI, Basel, Switzerland. This article is an open access article distributed under the terms and conditions of the Creative Commons Attribution (CC BY) license (http://creativecommons.org/licenses/by/4.0/).

E7 protein

```
atgcatggagatacacctacattgcatgaatatatgttagatttgcaaccagagacaact
 M  H  G  D  T  P  T  L  H  E  Y  M  L  D  L  Q  P  E  T  T
gatctctactgttatgagcaattaaatgacagctcagaggaggaggatgaaatagatggt
 D  L  Y  C  Y  E  Q  L  N  D  S  S  E  E  E  D  E  I  D  G
ccagctggacaagcagaaccggacagagcccattacaatattgtaacctttgttgcaag
 P  A  G  Q  A  E  P  D  R  A  H  Y  N  I  V  T  F  C  C  K
tgtgactctacgcttcggttgtgcgtacaaagcacacacgtagacattcgtactttggaa
 C  D  S  T  L  R  L  C  V  Q  S  T  H  V  D  I  R  T  L  E
gacctgttaatgggcacactaggaattgtgtgccccatctgttctcagaaaccataa
 D  L  L  M  G  T  L  G  I  V  C  P  I  C  S  Q  K  P  -
```

(**A**)

E7 protein - H2P (CAT → CCT)
- C24G (TGT → GGT)
- E46A (GAA → GCA)

(**B**)

```
E7wt        MHGDTPTLHEYMLDLQPETTDLYCYEQLNDSSEEEDEIDGPAGQAEPDRAHYNIVTFCCK 60
E7mutant    MPGDTPTLHEYMLDLQPETTDLYGYEQLNDSSEEEDEIDGPAGQAAPDRAHYNIVTFCCK 60
            * ********************* ********************* **************

E7wt        CDSTLRLCVQSTHVDIRTLEDLLMGTLGIVCPICSQKP- 98
E7mutant    CDSTLRLCVQSTHVDIRTLEDLLMGTLGIVCPICSQKP- 98
            **************************************
```

(**C**)

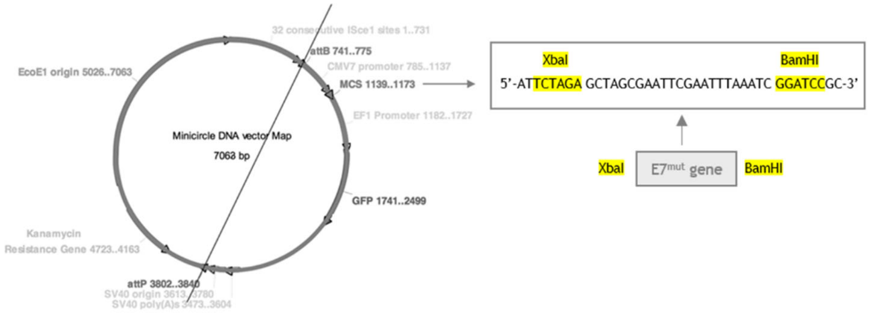

(**D**)

Figure S1. Schematic representation of three mutations int the HPV E7 gene and cloning of mutated gene in the parental plasmid vector. (**A**) E7 gene sequence from HPV wildtype; (**B**) Identification of three mutations to perform in the HPV E7 wildtype gene; (**C**) Sequencing of E7 mutant gene; (**D**) cloning of E7 mutant gene in the parental plasmid vector.

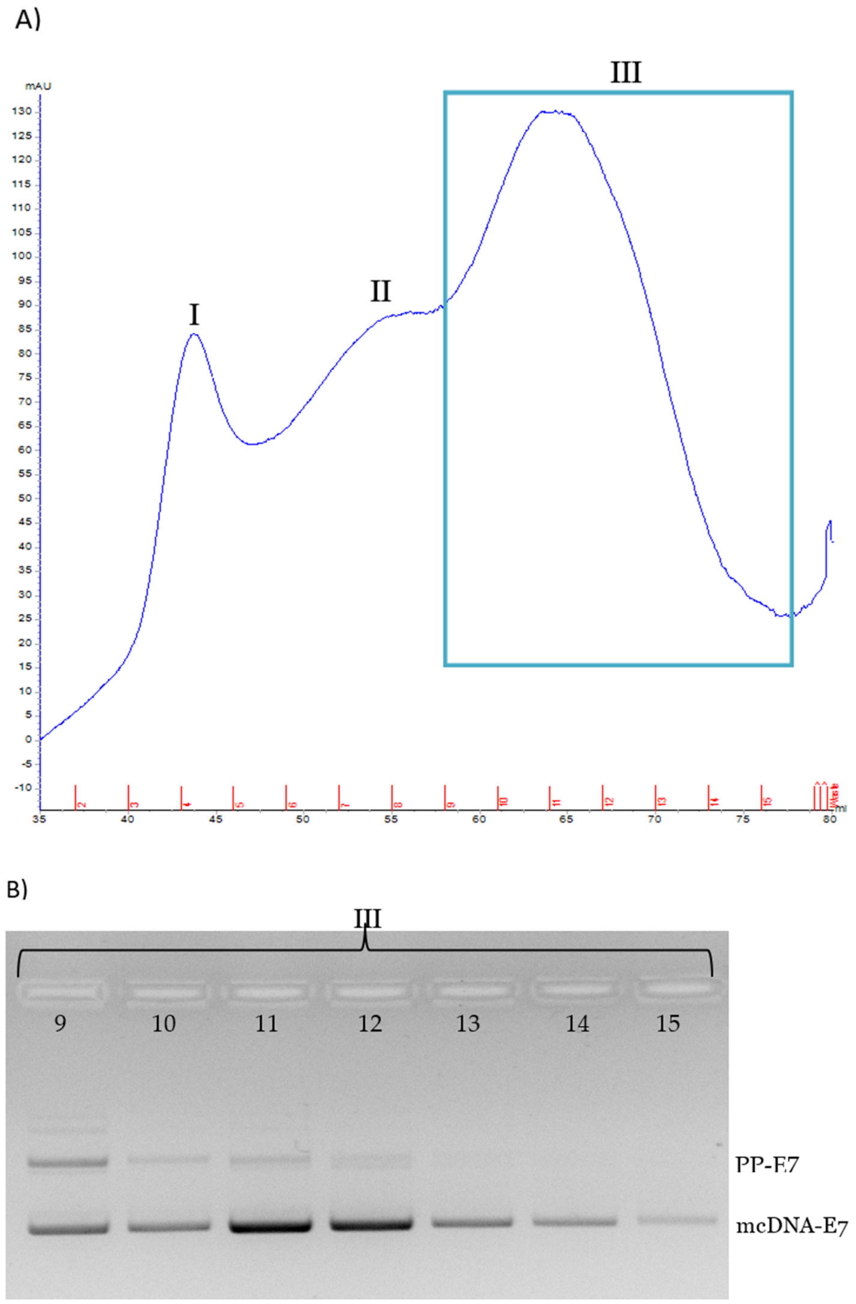

Figure S2. Chromatographic profile of mcDNA isolated by size exclusion chromatography in the Sephacryl SF-1000 column (**A**), using the following conditions, flow-rate of 0.3 mL/min, sample loading of 2 mL and fractionation of 3 mL; and agarose gel electrophoresis of fractions from peak III (**B**).

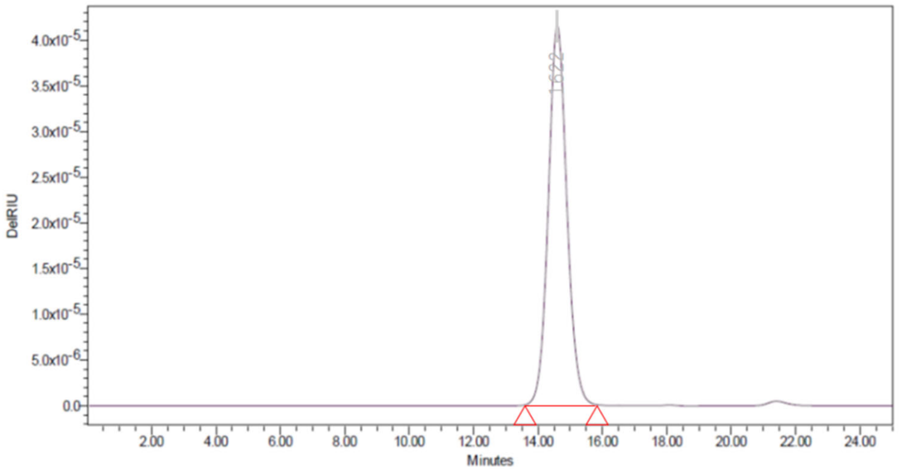

Figure S3. SEC chromatogram of MPITC-R8.

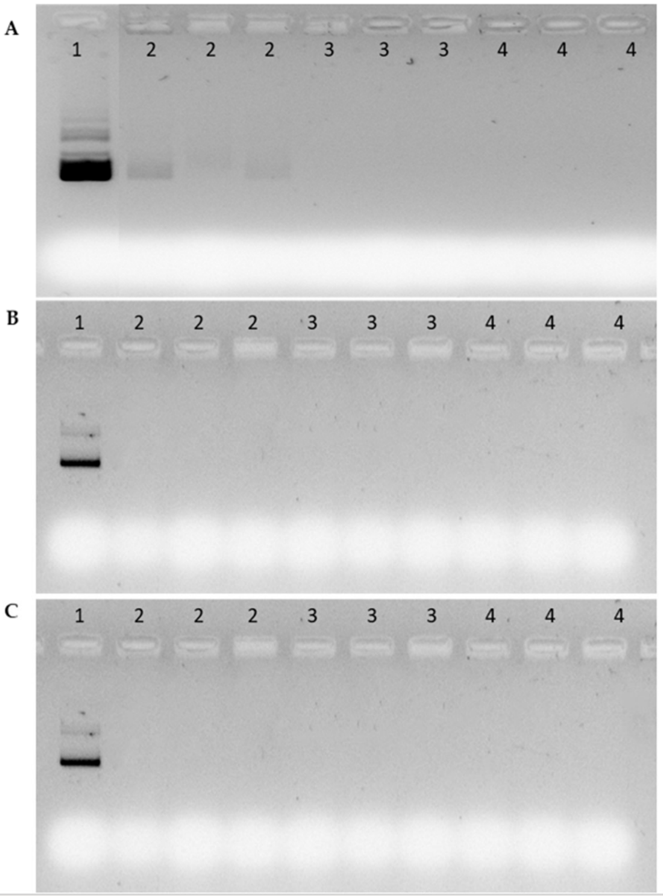

Figure S4. Electrophoretic mobility of supernatants from several formulations studied at various N/P ratios. (**A**) R8-mannose/mcDNA, (**B**) R8-mannose/PEI/mcDNA maintaining PEI N/P ratio at 5 and changing R8 N/P ratios and (**C**) R8-mannose/PEI/mcDNA maintaining PEI N/P ratio at 10 and changing R8 N/P ratios. Image A: lane 1-R8-mannose/mcDNA N/P ratio of 1:1; lane 2-R8-mannose/mcDNA N/P ratio of 1.5:1; lane 3-R8-mannose/mcDNA N/P ratio of 2:1. Image B: lane 1-R8-mannose/PEI/mcDNA N/P ratio of 1:5:1; lane 2-R8-mannose/PEI/mcDNA N/P ratio of 1.5:5:1; lane 3-R8-mannose/PEI/mcDNA N/P ratio of 2:5:1. Image C: lane 1-R8-mannose/PEI/mcDNA N/P ratio of 1:10:1: lane 2-R8-mannose/PEI/mcDNA N/P ratio of 1.5:10:1; lane 3-R8-mannose/PEI/mcDNA N/P ratio of 2:10:1.

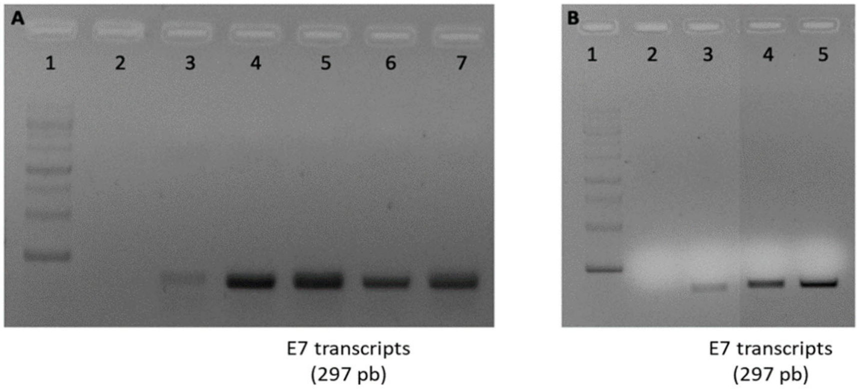

Figure S5. Analysis of RT-PCR products by agarose gel electrophoresis. Evaluation of E7 transcripts in Raw cells (**A**) and Fibro cells (**B**). Lane 1 - DNA molecular weight marker; lane 2-control without cDNA sample; lane 3-non-transfected cells; lane 4-cells transfected by PEI/mcDNA N/P ratio 5:1; lane 5-cells transfected by R8-mannose/PEI/mcDNA N/P ratio 2:5:1; lane 6-cells transfected by PEI/mcDNA N/P ratio 10:1; lane 7-cells transfected by R8-mannose/PEI/mcDNA N/P ratio 2:10:1.

Table S1. The molecular weight of each MPITC-R8 conjugate by SEC analysis.

Conjugates	Observed Relative Molecular Weight (Mw) (Da)	Theoretical Molecular Weight (Da)
MPITC-R8	1622	1643
